# Sex Differences Associated With Circulating PCSK9 in Patients Presenting With Acute Myocardial Infarction

**DOI:** 10.1038/s41598-018-35773-x

**Published:** 2019-02-28

**Authors:** Zhong Zhang, Teng-Fei Wei, Bei Zhao, Zhao Yin, Quan-Xing Shi, Pei-Lin Liu, Li-Feng Liu, Li Liu, Jing-Tao Zhao, Shuai Mao, Meng-Meng Rao, Shou-Li Wang, Yun-Dai Chen

**Affiliations:** 1 Department of Cardiology, 306th Hospital of PLA, Beijing, 100101 PR China; 2 Department of Cardiology, Chinese PLA General Hospital, Beijing, 100039 PR China

## Abstract

A limited number of studies have explored whether the role of circulating proprotein convertase subtilisin/kexin type 9 (PCSK9) in the pathogenesis of acute myocardial infarction (AMI) is sex specific. The purpose of the present study was to examine sex differences in plasma PCSK9 in Chinese patients with AMI. In this study, a total of 281 records from patients presenting with AMI were analyzed.We compared hospital data and plasma PCSK9 levels by sex difference for inpatients presenting with AMI. After 1 year of follow-up, major adverse cardiac events(MACE) were recorded. A Cox proportional hazards model was used to calculate hazard ratios with 95% confidence intervals. We found that, compared with male groups, PCSK9 levels were higher in female patients not only for overall patients with AMI but also for patients with ST-elevation myocardial infarction (STEMI) (median: 273.6 [215.6–366.8] vs. 325.1 [247.5–445.3] ng/ml, P = 0.0136; 273.4 [215.6–369.7] vs. 317.1 [249.6–450.1], P = 0.0275, respectively). The cumulative incidence of cardiac death and 1-year MACE were significantly higher in the female group compared with male group (10% vs. 2.74%, P = 0.025; 15% vs. 4.11%, P = 0.0054, respectively). On multivariate Cox regression analysis, female sex, total triglyceride, glycosylated hemoglobin A, and homocysteic acid were independent risk factors of 1-year MACE. There was no significant correlation between PCSK9 and 1-year MACE in total AMI patients. In conclusion, PCSK9 levels and 1-year MACE were higher in women with AMI than in men with AMI, however, female sex but not PCSK9 were significant correlated with the 1-year MACE. The clinical implications of this finding are worthy of further investigations and must be confirmed in larger cohorts.

## Introduction

Proprotein convertase subtilisin/kexin type 9 (PCSK9) has gained considerable attention over the past decade due to its role in elevating plasma levels of low density lipoprotein cholesterol (LDL–C), a major causal risk factor of coronary artery disease (CAD), by promoting the degradation of LDL receptors (LDL-R) in the liver^1,2^. Subsequently, a growing body of discoveries formed a clear association between PCSK9 function and cardiovascular risk in genetic^3–6^, experimental^7,8^, and epidemiologic data^9,10^.

A number of studies have suggested that a high level of plasma PCSK9 predicts future risk of cardiovascular events independently of established risk factors in the general population^11^ (over 60 years old) and patients with stable coronary artery disease (SCAD)^9,10,12^. However, few studies have comprehensively evaluated the association of plasma PCSK9 with the pathogenesis of acute myocardial infarction till now. As an important factor regulating cholesterol homeostasis, a high level of plasma PCSK9 has been observed in patients with acute myocardial infarction (AMI)^13^, a result which was confirmed in a rats model^14^. However, Liu’s^15^ study yielded conflicting results, finding that plasma levels of PCSK9 were significantly lower in patients with AMI compared to those with SCAD (290.42 ± 79.05 ng/ml vs.334.99 ± 85.96 ng/ml, P = 0.01). Whereas total PCSK9 concentration in the circulation is reportedly influenced by common and rare PCSK9 gene variants^16,17^, sex^18^, use of statins^19,20^, and diurnal variation^21^, it remains unknown whether or not the PCSK9 expression is influenced by the impact of AMI.

Furthermore, animal and human studies have shown that PCSK9 is also controlled by hormones such as estrogen^22,23^, growth hormone^22,24^, and insulin^25,26^. Since the pathogenesis of AMI is multifactorial, whether plasma PCSK9 have a gender specific approach remains unclear. Considering that difference in AMI risk factors between women and men, the aim of this retrospective cohort study was to examine sex differences in plasma PCSK9 in patients with AMI.

## Methods

The study complied with the Declaration of Helsinki and was approved by the hospital’s ethical review board (306th Hospital of PLA, Beijing, China), and all patients provided written informed consent.

### Population

A total of 342 patients were recruited between September 2013 and December 2015, with definite time of onset of acute MI and who underwent primary PCI within 24 h of onset. Acute MI was defined as ischemic symptoms lasting ≥30 min with ST-segment elevation or depression (≥1 mm) and elevated cardiac troponin I ≥ 0.03 ng/mL (non-ST elevation myocardial infarction, NSTEMI; ST-elevation myocardial infarction, STEMI). Inclusion criteria were as follows: (1) having a detailed clinical, laboratory data and well documented traditional cardiovascular risk factors; (2) underwent coronary angiography. Exclusion criteria were subjects over 90 years, pregnancy or lactation, psychiatric disorder, the existence of any infectious or systematic inflammatory disease within 1 month, serious heart failure or arrhythmia, significant hematologic disorders, thyroid dysfunction, severe liver dysfunction (aspartate aminotransferase or alanine aminotrabsferase three times more than the upper normal limits) and/or renal insufficiency (blood creatinine >1.5 mg/dL) and malignant tumors. Based on these criteria, 61 patients were excluded from the study. The remaining 281 patients were divided into 2 groups (male n = 220, female n = 61) according to sex difference, including 173 STEMI patients (male n = 135, female n = 38) and 108 NSTEMI patients (male n = 85, female n = 23).

### Definition of Conventional Cardiovascular Risk Factors

Hypertension was defined as repeated blood pressure measurements ≥140/90 mmHg (at least two times in different environments) or currently taking antihypertensive drugs. Diabetes mellitus (DM) was defined as fasting serum glucose level ≥ 7.0 mmol/L in multiple determinations, and/or the current use of medication for diabetes. Dyslipidemia was defined by medical history or the use of lipid-modulating medications in order to reduce lipids or fasting total cholesterol (TC) ≥ 200 mg/dL or triglyceride (TG) ≥ 150 mg/dL. Body mass index (BMI) was calculated as weight (kg) divided by height (m) squared, and obesity was defined as a BMI of ≥30 kg/m^2^. Patients with a reported smoking habit of at least one cigarette per day on admission were classified as current smokers.

### Measurement of PCSK9

Blood samples were drawn from the arterial sheath prior to coronary angiography and were stored at a temperature of −80 °C within 2 h after blood collection. Plasma PCSK9 concentrations were measured in the stored plasma samples using a quantitative sandwich enzyme immunoassay ELISA (catalog number Circulex CY-8079; CycLex Co., Ltd., Japan), according to manufacturer’s instructions. Concentrations of PCSK9 levels are given in nanograms per milliliter.

### Follow-Up

Patient follow-up was scheduled at 6 months, 1 year and annually thereafter using telephone and/or interview after the initial appointment by trained nurses or cardiologists. When clinical events were confirmed via telephone interview, we then contacted the patients’attending physician by mail.

Main clinical outcome measures were cardiac death, stroke, recurrent acute myocardial infarction (MI), and target vessel revascularization (TVR). Cardiac death was primarily confirmed by the death from the cardiac causes including sudden cardiac death, congestive heart failure, acute MI, severe arrhythmia, stroke, or other structural/functional cardiac diseases. Stroke was defined as acute cerebral infarction on the basis of the imaging or typical symptoms. Recurrent acute MI was diagnosed by a comprehensive evaluation combining chest pain or equivalent symptom complex, the diagnostic changes in cardiac enzyme levels, and the electrocardiogram. TVR was defined as repeat PCI for vessels successfully dilated at first admission. Major adverse cardiac events (MACE) were defined as a composite of individual clinical outcomes.

### Statistical analysis

The statistical analysis was performed with SPSS version 19.0 software (SPSS Inc, Chicago, IL). A P value < 0.05 was considered statistically significant.

The values were expressed as the mean ± SD or median (Q1–Q3 quartiles) for the continuous variables and the number (percentage) for the categorical variables. The differences of clinical and biochemical parameters between groups were analyzed using independent sample *t* test, Mann–Whitney *U* test, Chi-square tests, and Fisher’s exact test where appropriate. Linear regression analyses were performed to evaluate the associations of plasma PCSK9 levels with biomarkers in different sex. Multiple Cox regression analysis using forward elimination procedure adjusted for clinical risk factors was performed to identify independent predictors of MACE. The cumulative incidence of cardiac death and MACE were calculated using the Kaplan-Meier method and compared using the log-rank test. The results are presented as hazard ratios (HR) with 95% confidence interval (95% CI).

## Results

### Baseline Characteristics

The patient characteristics are described in Table 1. Compared with the male groups, female patients were older and more likely to have a lower rate of current smoking, but higher rates of comorbidities, such as hypertension and diabetes mellitus (*P* < 0.05). TC, HDL-C, HbA1c, BNP levels and prior CCB treatment were higher in female patients, but uric acid and homocysteic acid levels were lower in female patients. There were no statistically significant differences between the 2 groups in terms of BMI, obesity, dyslipidemia, family history of CAD, TG, LDL-C, glucose, hs-CRP, prior drug treatment (except CCB), and SYNTAX score.

**Table 1:** Baseline Characteristics in Patients with AMI.

	Male (n = 220)	Female (n = 61)	P value
Cardiovascular risk factors			
Age [years ($\bar{{\rm{x}}}$ ± s)]	58.53 ± 12.83	68.64 ± 8.97	<0.001
Hypertension [n (%)]	41 (18.64)	28 (45.90)	<0.001
Diabetes mellitus [n (%)]	63 (28.64)	28 (45.90)	0.011
Current smoking [n (%)]	162 (73.64)	4 (7.56)	<0.001
BMI [Kg/m^2^ ($\bar{{\rm{x}}}$ ± s)]	25.62 ± 3.50	24.87 ± 3.75	0.155
Obesity [n (%)]	41 (18.64)	13 (21.31)	0.639
Dyslipidemia [n (%)]	120 (54.55)	31 (50.82)	0.606
Family history of CAD [n (%)]	61 (27.73)	13 (21.31)	0.314
Biomarkers			
TC [mmol/l, M (Q1, Q3)]	4.30 (3.70,5.10)	4.75 (3.88,5.35)	0.045
TG [mmol/l, M (Q1, Q3)]	1.20 (0.80,1.70)	1.20 (0.80,1.90)	0.578
HDL-C [mmol/l, M (Q1, Q3)]	1.29 (1.14,1.47)	1.39 (1.25,1.62)	0.007
LDL-C [mmol/l, M (Q1, Q3)]	2.48 (1.94,3.13)	2.57 (2.08,3.23)	0.236
Glucose [mmol/l, M (Q1, Q3)]	6.58 (5.06,8.71)	7.1 (5.70,10.30)	0.060
HbA1c [%, M (Q1, Q3)]	6.00 (5.55,7.00)	6.50 (5.80,7.60)	0.015
Hs-CRP [mg/L, M (Q1, Q3)]	1.96 (0.73,5.90)	2.17 (0.67,6.20)	0.973
Uric acid [umol/L, M (Q1, Q3)]	332.5 (270.8, 399.5)	299 (238, 337)	0.001
Hcy [umol/L, M (Q1, Q3)]	12.30 (10.35,16.25)	9.60 (7.70,12.83)	<0.001
BNP [pg/ml, M (Q1, Q3)]	276 (117,754)	596 (184.5,1755)	0.002
Prior drug treatment			
Statin [n (%)]	42 (19.09)	11 (18.03)	0.852
Aspirin [n (%)]	23 (10.45)	7 (11.48)	0.819
Beta-blocker [n (%)]	13 (5.91)	4 (6.56)	0.851
CCB [n (%)]	42 (19.09)	24 (39.34)	0.001
ACEI/ARB [n (%)]	33 (15.00)	12 (19.67)	0.379
Coronary severity scores			
SYNTAX score	15 (9,21.5)	13.5 (8,19.5)	0.286

BMI, body mass index; TC, total cholesterol; TG, total triglyceride; HDL-C, high-density lipoprotein cholesterol; LDL-C, low-density lipoprotein cholesterol; HbA1c, glycosylated hemoglobin A; Hs-CRP, high-sensitive-C-reactive-protein; Hcy, homocysteic acid; BNP, B-type natriuretic peptide; CCB, calcium-channel blocker; ACEI, angiotensin converting enzyme inhibitor; ARB, angiotensin receptor blocker.

### Proprotein convertase subtilisin/kexin type 9 plasma level in men and women

**Figure 1:**
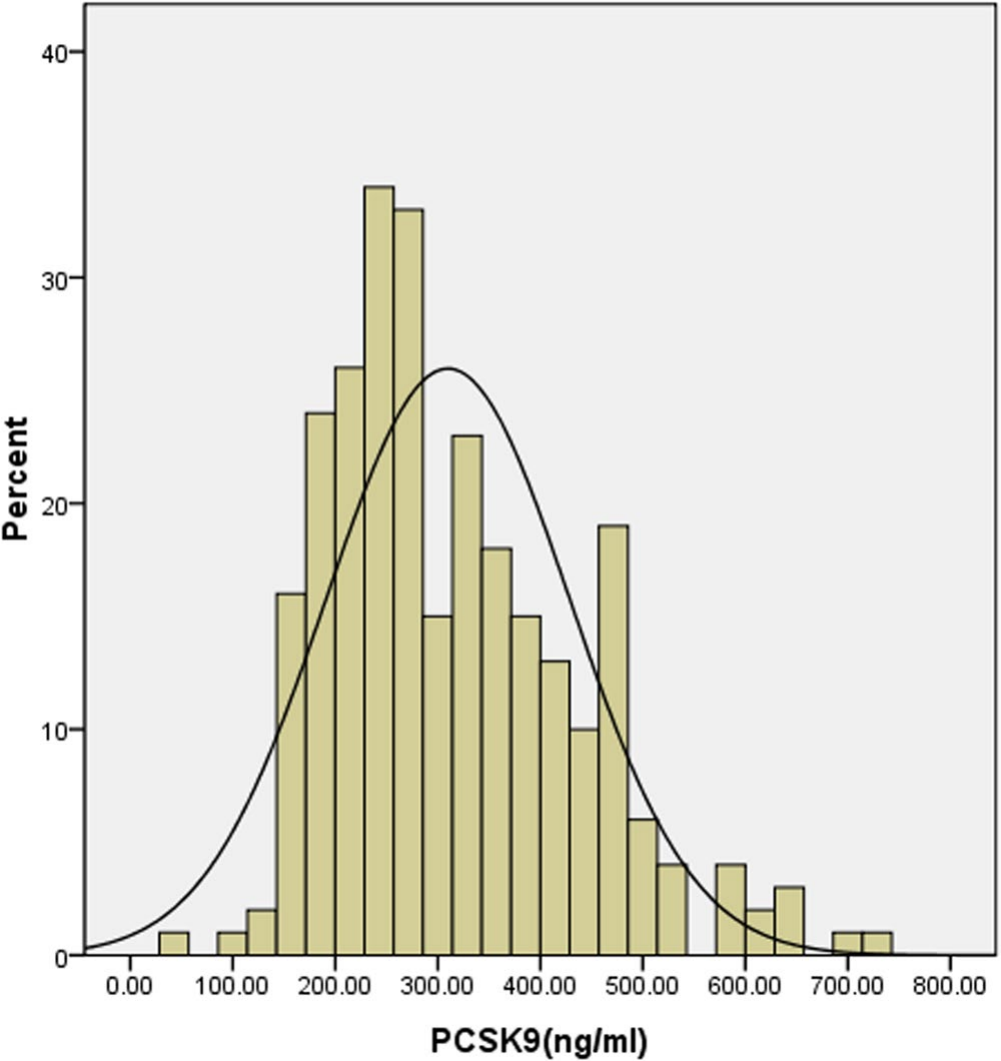
Distribution of plasma proprotein convertase subtilisin/kexin type 9 values in all AMI patients.

**Figure 2:**
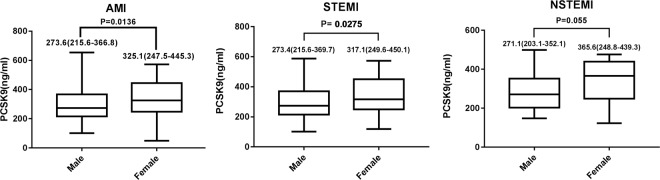
Proprotein convertase subtilisin/kexin type 9 plasma level in patients with AMI by sex Differences.

In total, median plasma PCSK9 level was 283.8 ng/ml and ranged from 49.22 to 730.8 ng/ml [interquartile range 227.7–393.3 ng/ml, Fig. 1]. Compared with male groups, PCSK9 levels were higher in female patients not only for overall patients admitted with AMI but also for patients with STEMI (median: 273.6 [215.6–366.8] vs. 325.1 [247.5–445.3] ng/ml, P = 0.0136; 273.4 [215.6–369.7] vs. 317.1 [249.6–450.1], P = 0.0275, respectively, Fig. 2). There were no statistically significant differences between the 2 groups for patients with NSTEMI (median: male 271.1 [203.1–352.1] vs. female 365.6 [248.8–439.3] ng/ml, P = 0.0552, Fig. 2).

### Correlation of proprotein convertase subtilisin/kexin type 9 plasma levels with other biomarkers in men and women

In both men and women, proprotein convertase subtilisin/kexin type 9 levels were not significantly correlated with lipid markers, such as total cholesterol, LDL cholesterol, HDL cholesterol, and total triglycerides (P > 0.05) (Table 2). No significant association was found between PCSK9 and cardiac troponin I, creatinine kinase MB, BNP, or hs-CRP in both groups (P > 0.05) (Table 2).

**Table 2:** Correlations between proprotein convertase subtilisin kexin 9 and other biomarkers.

Biomarkers	AMI (N = 281)		Male (N = 220)		Female (N = 61)	
	r	P	r	P	r	P
cTnI	−0.010	0.864	−0.008	0.911	0.005	0.969
CKMB	−0.032	0.592	−0.036	0.599	0.053	0.687
BNP	0.050	0.432	0.092	0.202	−0.210	0.124
Hs-CRP	−0.010	0.860	0.027	0.703	−0.228	0.091
TC	0.044	0.479	0.026	0.710	0.056	0.677
LDL-C	−0.025	0.681	−0.034	0.625	−0.009	0.948
HDL-C	0.038	0.536	0.045	0.518	−0.097	0.474
TG	0.041	0.504	0.069	0.320	−0.060	0.658

cTnI, cardiac troponin I; CKMB, MB isoenzyme of creatinine kinase; BNP, B-type natriuretic peptide; Hs-CRP, high-sensitive-C-reactive-protein; TC, total cholesterol; LDL-C, low-density lipoprotein cholesterol; HDL-C, high-density lipoprotein cholesterol; TG, total triglyceride.

### Clinical Outcome

One-year follow-up was available for (99.2%) of patients, and 18 episodes of MACE occurred during that time. Recurrent acute MI, TVR and stroke rates were similar between male and female patients (0.45% vs. 0, P > 0.99; 0.91% vs. 3.33%, P = 0.203; 0. vs. 1.67%, P = 0.215, respectively). The cumulative incidence of cardiac death and 1-year MACE were significantly higher in the female group compared with male group (10% vs. 2.74%, P = 0.025; 15% vs. 4.11%, P = 0.005, respectively, Table 3). Using multivariate Cox regression analysis using forward elimination, female sex, total triglyceride, glycosylated hemoglobin A, and homocysteic acid were found to be independent risk factors of 1-year MACE. There was no significant correlation between PCSK9 and 1-year MACE in total AMI patients (Table 4).

**Table 3:** The cumulative incidence of 1-year MACE in different sex.

AMI	Male (n = 219)	Female (n = 60)	P
Cardiac death, n (%)	6 (2.74)	6 (10.00)	0.025
Recurrent acute MI, n (%)	1 (0.45)	0	>0.99
TVR, n (%)	2 (0.91)	2 (3.33)	0.203
Stroke, n (%)	0	1 (1.67)	0.215
MACE, n (%)	9 (4.11)	9 (15.00)	0.005

TVR, target vessel revascularization; MACE, major adverse cardiac events.

**Table 4:** Multiple Cox regression analysis identify independent predictors for MACE.

	B	SE	P	HR	95% CI	
Age	−0.008	0.055	0.885	0.992	0.891	1.105
Female sex	1.430	0.665	0.031	4.180	1.136	15.374
Hypertension	−0.420	0.926	0.650	0.657	0.107	4.034
Diabetes mellitus	−1.918	1.508	0.203	0.147	0.008	2.823
Current smoking	1.181	1.371	0.389	3.257	0.222	47.806
Obesity	−2.083	2.493	0.997	0.008	0.000	1.121
Dyslipidemia	1.317	1.204	0.274	3.733	0.353	39.496
Family history of CAD	0.120	1.512	0.937	1.127	0.058	21.829
TG	0.026	0.009	0.003	1.027	1.009	1.044
TC	−0.031	0.236	0.895	0.969	0.610	1.540
HDL	1.513	1.830	0.408	4.541	0.126	16.977
LDL	−0.192	0.744	0.797	0.826	0.192	3.548
HbA1c	0.303	0.145	0.036	1.354	1.019	1.798
Hcy	0.036	0.015	0.013	1.037	1.008	1.067
PCSK9	0.000	0.002	0.967	1.000	0.996	1.004
Hs-CRP	−0.007	0.059	0.908	0.993	0.885	1.114
Prior Statin treatment	1.098	1.330	0.409	2.997	0.221	40.629
SYNTAX	0.014	0.046	0.763	1.014	0.926	1.111

TG, total triglyceride; TC, total cholesterol; HDL-C, high-density lipoprotein cholesterol; LDL-C, low-density lipoprotein cholesterol; HbA1c, glycosylated hemoglobin A; Hcy, homocysteic acid; PCSK9, proprotein convertase subtilisin/kexin type 9; Hs-CRP, high-sensitive-C-reactive-protein.

## Discussion

PCSK9 is known to elevate plasma levels of LDL–C by promoting the degradation of LDL receptors (LDL-R) in the liver^1,2^. Presently, PCSK9 inhibitors have been proven to significantly reduce LDL-C levels by 61% and reduce the incidence of cardiovascular events (CVEs) in the background of statin treatment during~1 year of therapy^27,28^. However, the relationship between circulating PCSK9 and pathophysiological mechanism of atherosclerosis is unknown. Leander *et al*.^11^ found that serum PCSK9 concentration is associated with incident CVD in 60-year-old individuals without prevalent CVD and that the association persists after adjustment for established CVD risk factors after 15 years of follow-up. However, another study^29^ has given conflicting result, casting doubt on association of PCSK9 levels with CVD. Several lines of evidence have indicated a positive correlation between circulating concentrations of PCSK9 and major adverse cardiovascular (CV) events in patients with stable CHD^9,10,12^ and acute coronary syndromes (ACS)^30^. Moreover, some studies have shown that PCSK9 maybe could be a biomarker for the severity of coronary artery disease^31^. However, it should be acknowledged that high initial PCSK9 plasma levels could not predict mortality at 1 year in ACS patients^13,30^.

Nevertheless, the role of circulating PCSK9 in patients with AMI remains unknown. In some clinical studies, a high level of plasma PCSK9 has been observed in patients with acute myocardial infarction (AMI)^13^, a result which has been further confirmed in a rats model^14^. However, Liu’s^15^ study found the conflicting results, which plasma levels of PCSK9 were significantly lower in patients with AMI compared with patients with SCAD (290.42 ± 79.05 ng/ml vs.334.99 ± 85.96 ng/ml, P = 0.01). Although clinical implications of the elevated plasma PCSK9 concentration in the acute period of AMI are still vague, mounting evidence suggests that PCSK9 has adverse effects on coronary plaques through several pathways, including proinflammatory LDL oxidation and direct modification of plaque composition^32–35^. Some studies have shown a positive correlation between circulating concentrations of PCSK9 and the fraction and amount of necrotic core tissue in coronary plaque by IVUS-VH imaging^36^. PCSK9 is also associated with increased oxidized LDL-induced apoptosis of human endothelial cells, which may lead to endothelial dysfunction and rupture of a thin-cap fibroatheroma lesion^34^. On the other hand, PCSK9 antibodies could stabilize the coronary plaque by decreasing macrophages and necrotic core content and increasing inflammatory monocyte recruitment^8^. PCSK9 is associated with an inflammatory response that is largely based on nuclear factor-κB-mediated expression of proinflammatory genes, including cytokines, chemokines, and adhesion molecules^33,37^. Furthermore, the PCSK9-induced nuclear factor-κB pathway can enhance the thrombotic substrate in atherosclerotic plaques through upregulation of tissue factor^38,39^. In aggregate, these data suggest that the increased plasma PCSK9 levels may be a possible trigger of atherosclerotic plaques destabilization.

Several lines of evidence have indicated a positive correlation between circulating concentrations of PCSK9 and LDL-C^2,17^. This association has not, however, been detected in the present study, which maybe due to the acute period of AMI. Myocardial infarction (MI) causes a rapid decline in LDL cholesterol levels^40^, making their correlation between circulating concentrations of PCSK9 and LDL-C be unreliable for the steady-state levels. Moreover, Kosenko’s^41^ study showed that LDL binds to PCSK9 and protects against PCSK9-mediated LDL receptor degradation, which might be possible interpretation for our study.

It is noteworthy that total PCSK9 concentration in the circulation is reportedly influenced by sex, and several studies have reported that PCSK9 is also controlled by hormones such as estrogen. Cui Q *et al*.^18^ have reported that serum PCSK9 levels are higher in postmenopausal women than in premenopausal women in a Han Chinese population. Several studies have indicated that circulating PCSK9 levels are inversely correlated with estrogen levels in healthy volunteers (age 20–85 years)^22,23^, and the mean levels of PCSK9 were 10% higher in females than in males (P < 0.05). PCSK9 levels were 22% higher in postmenopausal than in premenopausal (P < 0.001) females. Thus, aforementioned findings suggesting the sex difference of circulating PCSK9. In the present study, we found that, compared with male groups, PCSK9 levels were higher in women for both all admitted patients and specifically for patients with STEMI (median: 273.6 [215.6–366.8] vs. 325.1 [247.5–445.3] ng/ml, P = 0.0136; 273.4 [215.6–369.7] vs. 317.1 [249.6–450.1] ng/ml, P = 0.0275, respectively). There were no significant differences between the 2 groups for patients with NSTEMI (P = 0.0552). Furthermore, the rate of cardiac mortality and MACEs in female patients were higher than male patients after 1year follow-up (P = 0.025, P = 0.0054, respectively).However, Cox regression analysis suggests that that 1year MACEs were significant correlated with female sex, total triglyceride, glycosylated hemoglobin A, and homocysteic acid, but not with PCSK9 in total AMI patients. This finding is inconsistent with previous results of positive correlation between PCSK9 and major adverse cardiovascular, and we speculate that it may be related with the following factors. Firstly, the relatively small sample size from a single center may have led to an underestimation of the association between PCSK9 and clinical outcome. Secondly, it is possible plasma PCSK9 levels are elevated as a consequence of acute MI (as an acute phase reactant), because *in vivo* models demonstrated that hepatic PCSK9 expression is enhanced in the context of MI at 12 to 96 hours, with a peak at 48 hours^14^. Finally, plasma PCSK9 levels were influenced by systemic inflammation, which is a pivotal factor in AMI progression and exacerbation. In mice, endotoxin elevates plasma PCSK9 levels^42^ and plasma PCSK9 levels might be similarly elevated in humans after an acute infection and contribute to increased risk of subsequent MI. In general, we did not find a correlation between circulating concentrations of PCSK9 and 1 year MACE in the present study, which maybe due to the prognosis of acute myocardial infarction is multifactor influenced. Although a positive correlation between circulating concentrations of PCSK9 and major adverse cardiovascular (CV) events in patients with stable CHD have indicated by several lines of evidence, the potential of PCSK9 in the prediction of major adverse cardiovascular events in patients with AMI in particularly based on sex differences is requires further prospective investigation.

Some limitations of this study need to be acknowledged. Firstly, male and female groups (mostly post-menopausal women) are inhomogeneous with regard to age and additional comorbidities present, which may be due to the relatively small lmited sample size from a single center. Furthermore, we did not exclude patients with prior statin treatment, which may have biased the prognostic value of PCSK9 levels, given the known association between use of statins and elevated in PCSK9 levels^20,43^.

Future more specific studies may be needed to check if PCSK9 levels maintain the same sex association with a cohort non-treated with statins. Secondly, as with all biomarker studies, pre-analytical (long-term stability of PCSK9 at −80 °C is poorly known) as well as analytical aspects may have affected our findings. As samples were stored for 2–3 years prior to analysis, degradation of PCSK9 may have occurred. It would be of great necessary establishing an analytical methodology to measure the stability of the PCSK9 in order to confirm and better understand its role in MACE in further research. Thirdly, the follow-up duration of the study is short and maybe contribute to the small number of outcomes. Finally, our study investigated the relationship of PCSK9 and clinical outcome according to sex difference in AMI rather than stable CAD, whether the PCSK9 expression is influenced by the impact of AMI remains unknown.

## Conclusions

In summary, in the present study, we found PCSK9 levels and 1-year MACEs were higher in female patients than male not only for overall patients admitted with AMI but also specifically in STEMI, however, female sex but not PCSK9 were significant correlated with the1-year MACEs. The clinical implications of this finding are worthy of further investigations and must be confirmed in larger cohorts.
